# Myeloid Differentiation Factor 88 (MyD88)-Deficiency Increases Risk of Diabetes in Mice

**DOI:** 10.1371/journal.pone.0012537

**Published:** 2010-09-02

**Authors:** Toru Hosoi, Shota Yokoyama, Suguru Matsuo, Shizuo Akira, Koichiro Ozawa

**Affiliations:** 1 Department of Pharmacotherapy, Graduate School of Biomedical Sciences, Hiroshima University, Hiroshima, Japan; 2 Department of Host Defense, Research Institute for Microbial Diseases, Osaka University, Osaka, Japan; Louisiana State University, United States of America

## Abstract

**Background:**

Multiple lines of evidence suggest innate immune response pathways to be involved in the development of obesity-associated diabetes although the molecular mechanism underling the disease is unknown. Recent observations suggest that saturated fatty acids can act as a ligand for toll-like receptor (TLR) 4, which is thought to mediate obesity-associated insulin resistance. Myeloid differentiation factor 88 (MyD88) is an adapter protein for TLR/IL-1 receptor signaling, which is involved in the activation of inflammatory pathways. To evaluate molecular mechanisms linking obesity-associated diabetes down-stream of TLR4, we investigated physiological role of MyD88 in high-fat diet (HFD)-induced obesity.

**Methodology/Principal Findings:**

In the present study, we found MyD88-deficient mice fed a HFD had increased circulating levels of insulin, leptin and cholesterol, as well as liver dysfunction (increased induction of ALT levels, increased activation of JNK and cleavage of PARP), which were linked to the onset of severe diabetes. On the other hand, TNF-α would not be involved in HFD-induced diabetes in MyD88-deficient mice, because TNF-α level was attenuated in MyD88-deficient mice fed with HFD.

**Conclusions/Significance:**

The present finding of an unexpected role for MyD88 in preventing diabetes may provide a potential novel target/strategy for treating metabolic syndrome.

## Introduction

Multiple lines of evidence suggest innate immune response pathways to be involved in the development of obesity-associated diabetes. However, the molecular mechanism underling the disease is unknown [1].

Excess accumulation of fatty acids is a characteristic of metabolic disease. Toll-like receptor 4 (TLR4) is known to play a critical role in the activation of innate immune responses by recognizing lipopolysaccharide. Interestingly, recent observations suggest that saturated fatty acids can act as a ligand for TLR4 [2], [3]. TLR4 deficient/mutated mice have been shown to protect from obesity-associated insulin resistance [3]-[7]. Collectively, these findings indicate TLR4-mediated signaling would exacerbate metabolic syndrome by enhancing inflammation. However, the intracellular mechanisms of TLR4-mediated metabolic disease are unknown. Thus, in the present study, we focused on by possible functional role of myeloid differentiation factor 88 (MyD88), an essential adapter protein for TLR/interleukin (IL)-1 receptor signaling [8], [9], in metabolic disease. MyD88 is originally isolated as myeloid differentiation primary response gene, which is induced in M1 myeloleukemic cells in response to interleukin-6 [10]. Subsequently, MyD88 was found to be related to the interleukin-1 receptor (IL-1R) family including Toll/TLR protein, which is homologous to that of IL-1R [11]. Finally, it has been demonstrated that signaling via TLR4 employs MyD88 as an adaptor protein, that induces activation of NFκB through interleukin 1 receptor-associated kinase (IRAK) kinase and TNF receptor-associated factor 6 (TRAF6) [12]. Such activation is essential for the induction of innate immune responses.

The purpose of this study was to test the physiological role of MyD88 in the state of metabolic disease using MyD88-deficient mice. As saturated fatty acids-induced activation of TLR4 is involved in enhancing metabolic disease, MyD88-deficient mice would be expected to attenuate obesity-associated diabetes. However, contrary to expectations, we found that these mice exhibit severe diabetic phenotype.

## Results

### MyD88-deficiency exacerbates diabetes without affecting body weight or adiposity

**Figure 1:**
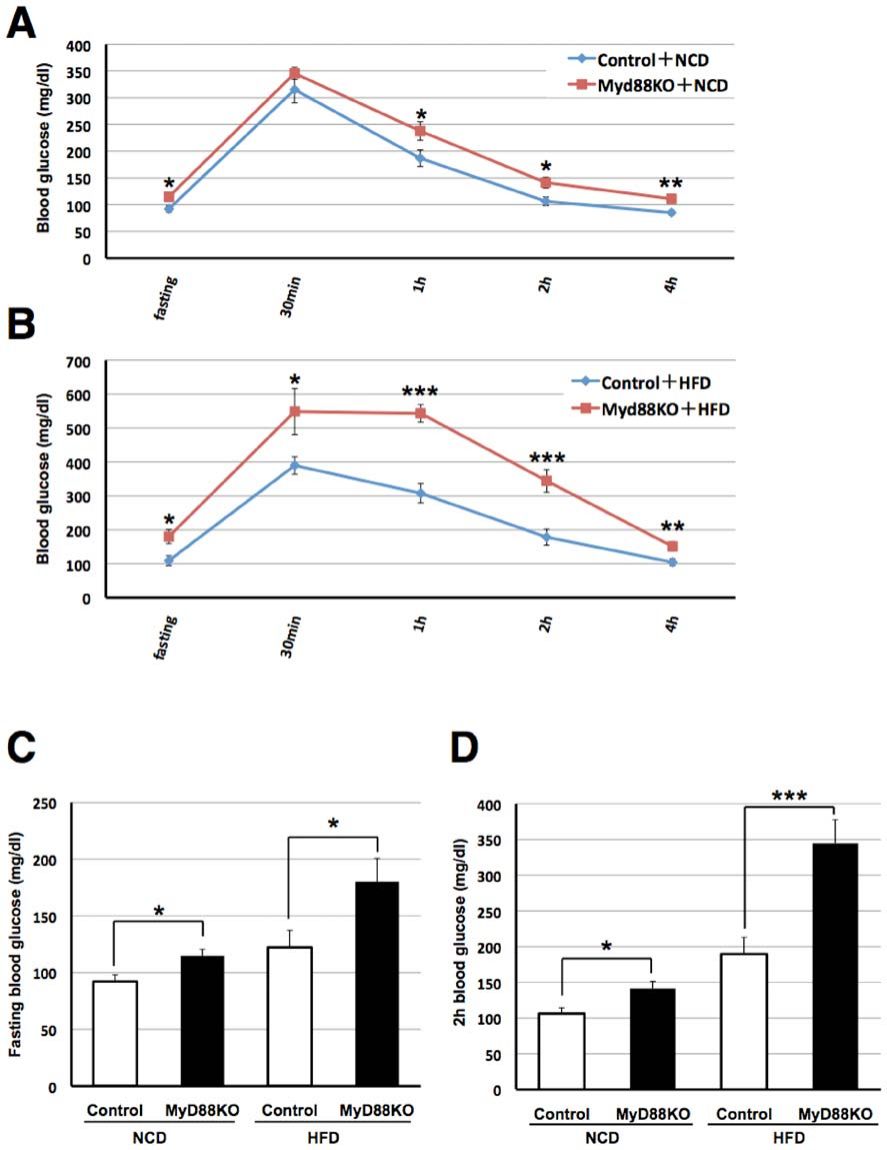
MyD88-deficiency increases the risk of diabetic mellitus. Both genotypes were fed a NCD until 6 weeks of age and then fed a NCD or HFD for 10 weeks. Mice were fasted for 16 h (from 18:00 to 10:00) and subjected to a glucose tolerance test (GTT). We measured circulating levels of glucose at the time indicated (0.5-4 h). (**A**) NCD-fed mice. (**B**) HFD-fed mice. (**C**) NCD- and HFD-fed mice, which were fasted for 16 h. (**D**) Glucose levels after GTT (2 h time point) of each genotype of mice fed the NCD or HFD. n = 6∼12/group. *p<0.05, **p<0.01, ***p<0.001 v.s. control mice.

**Figure 2:**
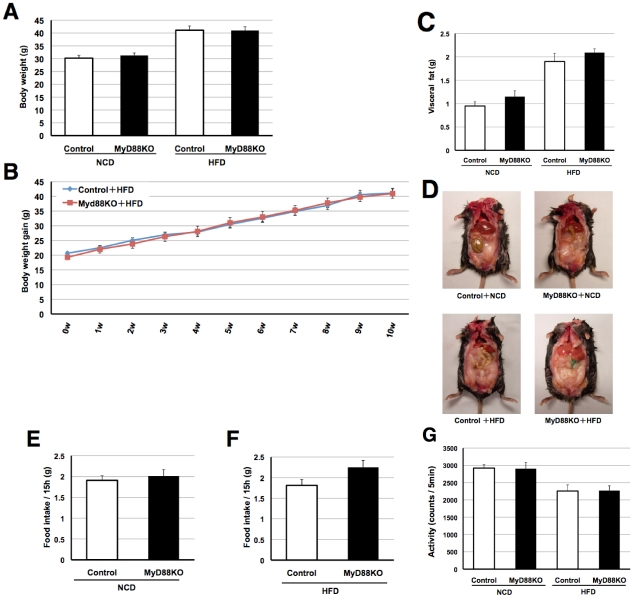
MyD88-deficiency did not affect body weight, adiposity, food intake or locomotor activities. (**A**) Body weight gain was measured in control and MyD88-deficient mice. Both genotypes were fed a NCD until 6 weeks of age and then fed a NCD or HFD for 10 weeks. NCD (n = 21–29) and HFD (n = 20–26)-fed mice were examined at the age of 16 weeks. (**B**) Control and MyD88-deficient mice were fed the HFD from the age of 6 weeks and body weight gain was measured every week (10 weeks). (n = 16–23) (**C**) Visceral fat was measured at the age of 18 weeks. NCD (n = 8) HFD (n = 6–8) (**D**) Typical photograph of each genotype of mice fed the NCD or HFD. (**EF**) Fifteen hours (From19:00 to 10:00) of food intake was measured in each genotype of mice fed the NCD or HFD at the age of 16 weeks. n = 11–13/group (**G**) Locomotor activities (5 min) were measured in each genotype of mice fed the NCD or HFD at the age of 16 weeks. n = 11–14/group.

**Figure 3:**
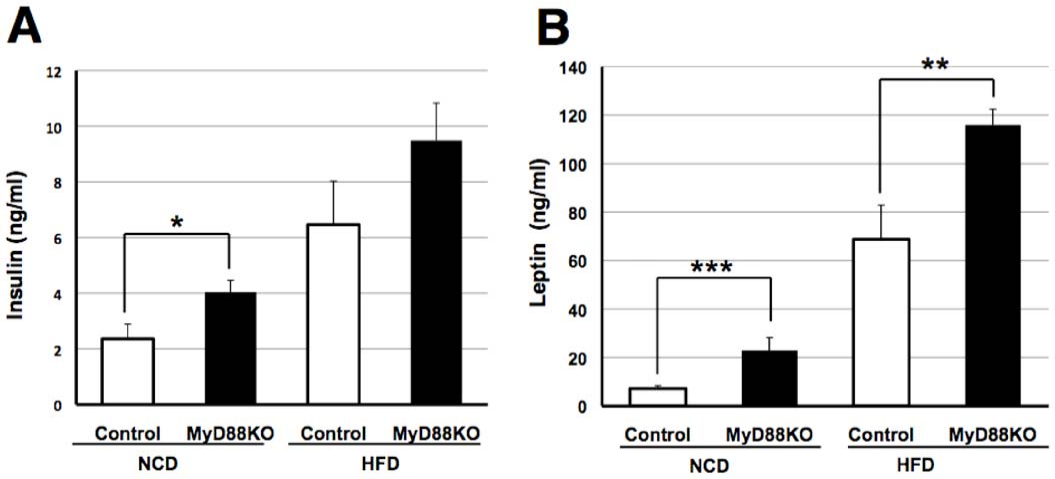
Increased circulating levels of insulin and leptin in MyD88-deficient mice. (**A**) Plasma insulin was measured in each genotype fed the NCD (n = 9–10) or HFD (n = 10–15) at the age of 16 weeks. *p<0.05 (**B**) Plasma leptin was measured in each genotype fed the NCD (n = 9–16) or HFD (n = 11–12) at the age of 16 weeks. **p<0.01, ***p<0.001.

To assess whether MyD88 is involved in glucose metabolism, we performed the glucose tolerance test (GTT) using age-matched systemic MyD88-deficient and control mice. First, we measured fasted blood glucose levels and found them to be increased in MyD88-deficient mice on either a normal chow diet (NCD) or a high-fat diet (HFD) (Figure 1A–D). In addition, GTT markedly raised blood glucose levels in MyD88-deficient mice compared to the controls (Figure 1A–D). The increase in blood glucose differed more between the MyD88-deficient and control mice fed the HFD (Figure 1A–D). However, no genotype-dependent differences in body weight gain (Figure 2AB) or visceral fat content (Figure 2CD) were observed in the NCD or HFD-fed state, suggesting that the difference in circulating glucose levels was not dependent on obesity. In addition, no differences in food intake (Figure 2EF) or locomotor activity (Figure 2G) were observed in either genotypes. These results are unexpected because impaired function/expression of TLR4 has been reported to protect against obesity-associated diabetes [3]–[5]. Consequently, we measured circulating levels of insulin and leptin by ELISA, and found them to be elevated in MyD88-deficient mice (Figure 3AB), suggesting type 2 diabetes.

### Increased circulating level of cholesterol and JNK activation in MyD88-deficient mice fed with HFD

**Figure 4:**
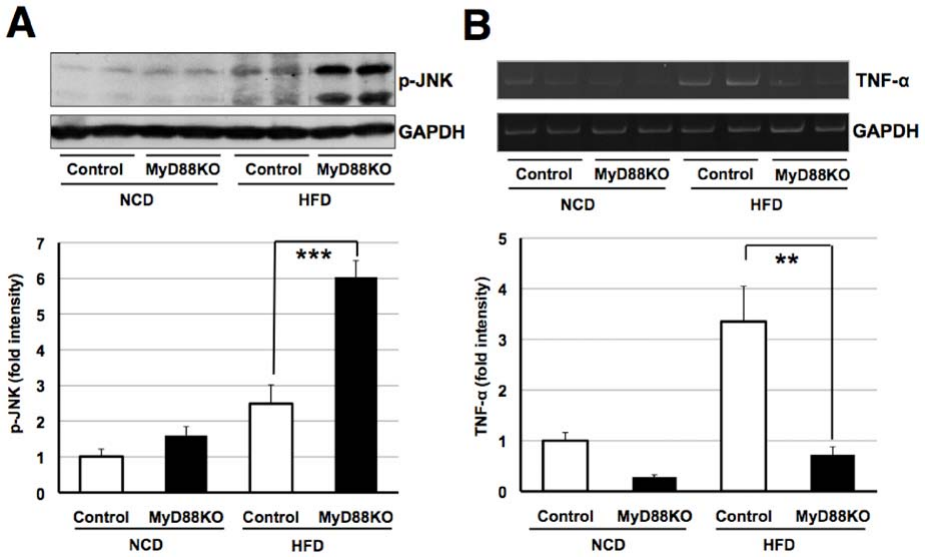
JNK activation without affecting TNF-α levels in MyD88-deficient mice fed the HFD. (**A**) Western blot analysis of p-JNK in liver samples at the age of 18 weeks. Each genotype of mice was fed the NCD (n = 7) or HFD (n = 6–8). ***p<0.001 (**B**) mRNA level of TNF-α was measured in liver samples at the age of 18 weeks. (n = 6–8) **p<0.01.

As the activation of c-Jun amino-terminal kinase (JNK) in the liver plays a critical role in the development of diabetes [13], [14], we next examined whether levels of phosphorylated JNK are altered in MyD88-deficient mice. Consistent with a previous report [14], we observed an increase in the phosphorylation of JNK in normal mice fed a HFD (Figure 4A). To our surprise, the increase was drastically enhanced in MyD88-deficient mice on the HFD (Figure 4A). The result was unexpected because MyD88-deficient mice are resistant to the LPS-induced activation of JNK [8].

**Figure 5:**
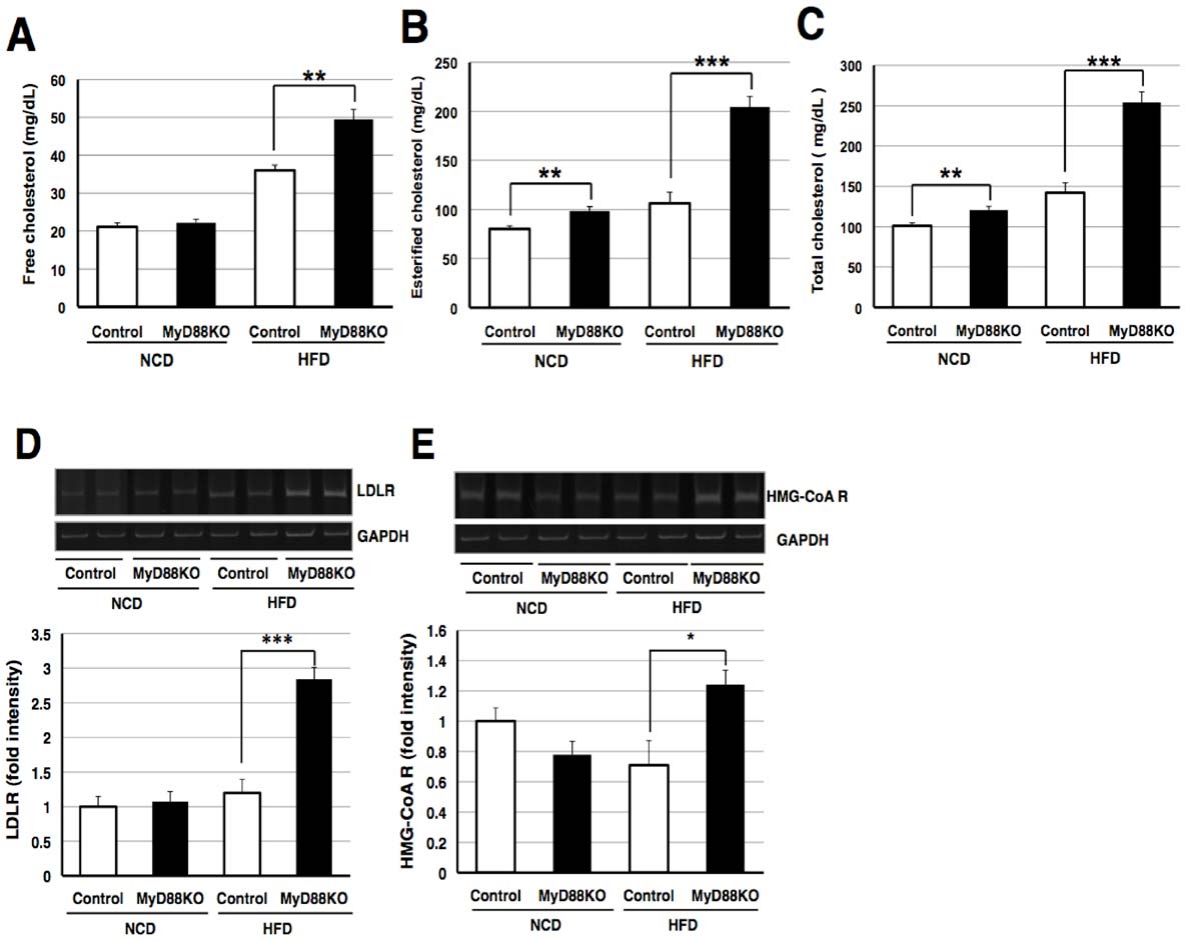
MyD88-deficient mice fed the HFD increases circulating level of cholesterol. (**A–C**) Serum cholesterol concentrations were measured at the age of 18 weeks. (**A**) Free cholesterol, (**B**) esterified cholesterol and (**C**) total cholesterol levels. Each genotype of mice was fed the NCD (n = 8) or HFD (n = 6–7). **p<0.01, ***p<0.001 (**DE**) mRNA levels of LDLR and HMG-CoA reductase (HMG-CoA R) were measured in liver samples at the age of 18 weeks. (n = 6–8) *p<0.05, ***p<0.001.

To identify the mechanisms linking JNK's activation in MyD88-deficient mice fed the HFD, we examined the level of tumor necrosis factor-α (TNF-α) in the liver, as TNF-αis involved in the activation [15]. The level of mRNA encoding TNF-αwas elevated in HFD-fed control mice (Figure 4B). However, this increase was markedly inhibited in MyD88-deficient mice on the HFD (Figure 4B). Thus, TNF-αexpression is induced via a MyD88-dependent pathway in mice fed a HFD. Moreover, TNF-αmay not be involved in activating JNK in such mice. To identify the factor responsible for activating JNK in MyD88-deficient mice, we next analyzed serum cholesterol levels because cholesterol can induce JNK's activation [16]. We observed an increase in cholesterol (total, free and esterified cholesterol) levels in HFD-fed normal mice and a marked increase in MyD88-deficient mice fed the HFD (Figure 5A–C). We also found that low density lipoprotein receptor (LDLR) was drastically up-regulated in the liver sample of MyD88-deficient mice fed the HFD (Figure 5D), suggesting positive feedback regulation against the increased circulating levels of cholesterols to remove them [17], [18]. Interestingly, transcriptional level of HMG-CoA reductase, the rate-limiting enzyme of cholesterol biosynthesis [19], was up-regulated in the liver sample of MyD88-deficient mice fed the HFD (Figure 5E). Thus, the increased level of cholesterol observed in theses mice would be due to the induction of HMG-CoA reductase. Moreover, these results suggest that the increased levels of cholesterol in MyD88-deficient mice to be responsible for the activation of JNK.

### Liver dysfunction in MyD88-deficient mice fed with HFD

**Figure 6:**
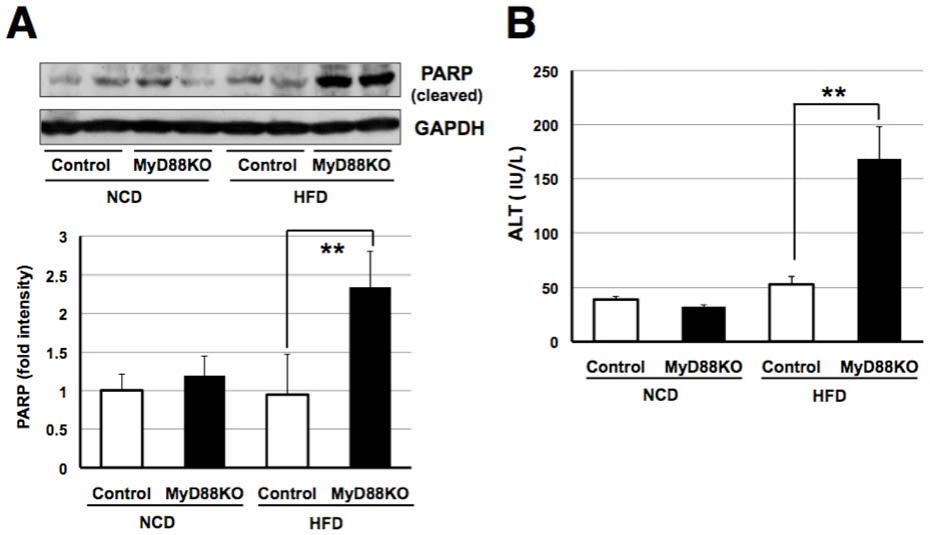
MyD88-deficient mice fed the HFD developed liver dysfunction. (**A**) Western blot analysis of cleaved PARP in liver samples at the age of 18 weeks. Each genotype of mice was fed the NCD (n = 7) or HFD (n = 6–8). **p<0.01 (**B**) Serum ALT levels were measured at the age of 18 weeks. Each genotype of mice was fed the NCD (n = 8) or HFD (n = 6–7). **p<0.01.

JNK's activation results in apoptosis [20]. Thus, to assess whether these differences would result in liver dysfunction, we next measured cleavage of poly (ADP-ribose) polymerase (PARP), an indicator of the apoptotic state, in liver samples of both genotypes of NCD and HFD-fed mice. As shown in Figure 6A, we found an increase in PARP cleavage in MyD88-deficient mice fed the HFD. Furthermore, the serum concentration of alanine aminotransferase (ALT), a marker of hepatic injury, was significantly elevated in MyD88-deficient mice fed the HFD compared with normal mice (Figure 6B). Together, these findings indicate that the diabetic phenotype observed in Myd88-deficient mice was due at least in part to increased apoptosis in the liver possibly mediated via hypercholesterolemia.

## Discussion

Increasing evidence indicate the link among obesity, diabetes and inflammatory pathways in developing metabolic diseases. As saturated fatty acids-induced activation of TLR4 is involved in enhancing metabolic disease, we expected that MyD88 would exacerbate metabolic disease. However, to our surprise, we found that MyD88 is involved in protecting against diabetes mellitus. We found that MyD88-deficient mice fed with high fat diet develop severe diabetes. From these observations, we are next interested in investigating mechanisms of diabetes observed in MyD88-deficiency. As TNF-αis involved in the development of insulin resistance [21]–[23] and that MyD88 is involved in activating inflammatory signals [8], [24], we measured liver TNF-αlevels in these mice. We found that HFD-induced induction of this cytokine was impaired in MyD88-deficient mice. Collectively these results indicate that TNF-αis not involved in diabetes observed in MyD88-deficient mice. Then, what is the responsible factor exacerbating diabetes in MyD88 deficiency? In the present study, we investigated possible involvement of cholesterol in the development of such phenotype and found that circulating cholesterol levels as well as liver LDLR and HMG-CoA levels were drastically increased in MyD88-deficient mice fed a HFD. Elevated levels of cholesterol will increase apoptosis [25]. We observed JNK activation and liver dysfunction (PARP cleavage and increase in ALT levels) in MyD88-deficient mice fed a HFD. Thus, these results suggest that cholesterol may be involved in diabetes observed in MyD88-deficient mice. It would be useful to analyze the double knockout mice for MyD88 and cholesterol related gene such as HMG-CoA reductase to conform the results more directly.

Intriguingly, the deletion of MyD88 restricted to the central nervous system had been reported to protect against HFD-induced impairment of glucose tolerance [26]. Taking into account the present contrasting finding that a systemic deficiency of MyD88 in mice exacerbated the HFD-induced impairment of glucose tolerance, we speculate that the physiological role of MyD88 differs among tissues expressed. Considering the role of liver on glucose homeostasis, the important role of liver JNK or IκB kinase-β in the development of diabetes has been reported [27]–[29]. On the other hand, the important role of central nervous system (CNS) on regulation of glucose homeostasis has been suggested [30]. Interestingly, although insulin action at CNS and peripherally can both reduce glucose levels, peripheral action of insulin is an anabolic (stimulates nutrient storage), which contrast with its catabolic function at CNS (inhibits food intake and stimulates energy expenditure)[31]. Thus, the metabolic action of CNS and peripheral organs would be different. It is therefore interesting subject to further analyze the pathophysiological role of MyD88 in the brain v.s. periphery. In addition, it will be needed to investigate what type of cell is responsible for diabetes observed in MyD88-deficiency. The use of conditional knockout mouse (such as liver-specific knockout mouse) would be useful to answer these questions. Importantly, MyD88-deficiency in human subjects has been reported [32]. At present, it is unknown whether MyD88-deficient human subjects are prone to diabetes and it will be an open questions.

The present results indicate an unanticipated important link between MyD88 and the development of diabetes mellitus. Our findings would provide a molecular basis for understanding diabetes and developing a novel pharmacological treatment targeting MyD88.

## Materials and Methods

### Animals

Adult male C57BL/6CrSlc mice (wild type, WT) and MyD88-deficient mice (MyD88 ^−/−^) mice were used in the present study. The MyD88-deficient mice (C57BL/6 Cr Slc back ground) were kindly provided by Dr. Shizuo Akira (Department of Host defense, Research Institute for Microbial Disease, Osaka University). The C57BL/6 Cr Slc control mice were obtained from SLC (Hamamatsu, Japan). Mice were maintained in a room at 22–24°C under a constant day-night rhythm and given food and water, ad libitum. They were fed either a normal chow diet (NCD: MF diet; Oriental Yeast, Tokyo, Japan) or a high-fat diet (HFD: D12492; Research diets, NJ). The NCD and HFD contained 5.3% and 35% fat, respectively. All animal experiments were carried out in accordance with the NIH Guide for Care and Use of Laboratory Animals and approved by the animal care and use committee at Hiroshima University (Permit number: A10-41).

### Glucose Tolerance Test

The Glucose Tolerance Test was performed with mice fasted 16 h (18:00–10:00). After measuring the fasted blood glucose level, we injected glucose (2 g/kg, 15 ml/kg) through the intraperitoneal route. Blood glucose levels were measured after 0.5, 1, 2 and 4 h using NIPRO Freestyle Freedom (Osaka, Japan).

### Body Weight

Body weight was measured once a week at 16:00–17:00.

### Food intake

Both genotypes of mice were housed individually prior to the experiment. Food intake was measured at the end of the study period of 16 weeks.

### Locomotor activities

An open field locomotor test was performed to monitor locomotor activities. Mice were placed at the centre of a cubic chamber (48×48×48 cm). The animal's horizontal movements (5 min) were measured by automatic actography (SCANET MV-10; Melquest, Toyama, Japan). The test room was dimly illuminated with indirect white lighting.

### Measurement of insulin and leptin levels

Blood samples (including EDTA) were taken by decapitation and centrifuged (4°C, 3,000 rpm, 15 min) to obtain plasma samples. Plasma insulin (Morinaga; Tokyo, Japan) and leptin (R & D systems; MN) levels were measured by ELISA according to the manufacturer's guidelines.

### Measurement of cholesterol and ALT levels

Blood samples were taken by decapitation and stood for 2 hours at room temperature. The samples were then centrifuged (4°C, 3,000 rpm, 15 min) to obtain serum. Serum cholesterol and ALT levels were measured at SRL, Inc. (Tokyo, Japan). Cholesterol levels were measured by HDAOS and DAOS methods. ALT levels were measured by the UV method.

### Preparation of liver samples

Mice were sacrificed by decapitation and the liver was quickly removed and rapidly dissected on an ice-cold plate. The samples were snap-frozen in liquid nitrogen and stored at −80°C prior to use.

### Western blot analysis

Western blotting was performed as described previously [33]. Liver samples were homogenized with 100 revolutions in a glass homogenizer containing 10 mM HEPES–NaOH (pH 7.5), 150 mM NaCl, 1 mM EDTA, 1 mM Na_3_VO_4_, 10 mM NaF, 10 µg/ml aprotinin, 10 µg/ml leupeptin, 1 mM PMSF and 1% NP-40. The samples were then centrifuged at 20,630 g for 45 min at 4°C and the supernatants were collected. Laemmli buffer was added to the samples and boiled for 3 min. The samples were fractionated by SDS-PAGE (100–200 µg/lane) and transferred at 4°C to nitrocellulose membranes. The membranes were blocked and incubated with an anti-phospho (Thr183/Tyr185)-JNK (Cell signaling; 1:1,000), anti-PARP (Santa Cruz Biotechnology; 1:1,000) or anti-GAPDH (Chemicon; 1:1,000) antibody at 4°C. The membranes were washed and then incubated with an anti-horseradish peroxidase-linked antibody (GE Healthcare). Peroxidase was detected using an ECL system (GE Healthcare). The density of bands was measured using Image J 1.37v (Wayne Rasband, NIH) software.

### Gene expression analysis

Tissue samples were homogenized at 10,000 rpm using a polytron homogenizer in TriPure Isolation Reagent (Roche Diagnostics) and total RNA was isolated according to the manufacturer's protocol. cDNA was synthesized from 2 µg of total RNA by reverse transcription using 25 U of Superscript Reverse Transcriptase III(Invitrogen) and 0.25 µg of Oligo(dt)12–18 primer (Invitrogen) in a 20-µl reaction mixture containing First-Strand Buffer (Invitrogen), 1 mM dNTP mix, 10 mM DTT, and 20 U of RNaseOUT Recombinant Ribonuclease Inhibitor (Invitrogen). Total RNA and the Oligo (dt) 12–18 primer were pre incubated at 70°C for 10 min prior to the reverse transcription. After incubation for 1.5 h at 46°C, the reaction was terminated by incubating samples for 15 min at 70°C.

For PCR amplification, 1.2 µl of cDNA was added to 10.8 µl of a reaction mix containing 0.2 µM of each primer, 0.2 mM of dNTP mix, 0.6 U of Taq polymerase (Roche Diagnostics), and reaction buffer. PCR was performed in a DNA Thermal Cycler (MJ Research, PTC-220). The following primer sequences were used: TNF-α; upstream, 5′–cac gtc gta gca aac cac caa-3′, and downstream, 5′-ccc att ccc ttc aca gag caa-3′, LDLR; upstream, 5′–tcc aat caa ttc agc tgt gg-3′, and downstream, 5′-gag cca tct agg caa tct cg-3′, HMG-CoA reductase; upstream, 5′–agc ttg ccc gaa ttg tat gtg-3′, and downstream, 5′-tct gtt gtg aac cat gtg act tc-3′, GAPDH; upstream, 5′-aaa ccc atc acc atc ttc cag-3′ and downstream, 5′-agg ggc cat cca cag tct tct-3′. The PCR products (10 µl) were resolved by electrophoresis in an 8% polyacrylamide gel in TBE buffer. The gel was stained with ethidium bromide and photographed under ultraviolet light. The density of bands was measured using Image J 1.37v (Wayne Rasband, NIH) software.

### Statistics

Results are expressed as the mean ± S.E.. Statistical analyses were performed using Student's *t*-test.
